# Characterizing Marine Medaka (*Oryzias melastigma*) Haploid Embryonic Stem Cells: A Valuable Tool for Marine Fish Genetic Research

**DOI:** 10.3390/ani14182739

**Published:** 2024-09-21

**Authors:** Wanwan Zhang, Huiquan Chen, Wei Liu, Kuntong Jia, Meisheng Yi

**Affiliations:** 1 School of Marine Sciences, Sun Yat-sen University, Guangzhou 519082, China; zhangww56@mail3.sysu.edu.cn (W.Z.); chenhq57@mail2.sysu.edu.cn (H.C.); liuw68@mail.sysu.edu.cn (W.L.); 2 Guangdong Provincial Key Laboratory of Marine Resources and Coastal Engineering, Guangzhou 519082, China; 3 State Key Laboratory of Biocontrol, Sun Yat-sen University, Guangzhou 519082, China

**Keywords:** haploid embryonic stem cells, marine medaka, pluripotency, viral susceptibility

## Abstract

**Simple Summary:**

Haploid embryonic stem cells (ESCs), with pluripotency and haploidy, hold significant value in the fields of developmental biology and reproductive technology. Here, the resources of haploid ESC line (hMMES1) from marine medaka, an emerging fish model for marine biology, were derived to address this need. The hMMES1 line was characterized with haploidy, pluripotency, transplantation ability, high edit effectivity, and viral susceptibility, marking a pioneering advancement as the next fish haploid ESCs after the Japanese medaka and, distinctively, as the first haploid ESCs within marine fish species.

**Abstract:**

Haploid embryonic stem cells (ESCs), which combine the properties of haploidy and pluripotency, hold significant potential for advancing developmental biology and reproductive technology. However, while previous research has largely focused on haploid ESCs in freshwater species like Japanese medaka (*Oryzias latipes*), little is known about their counterparts in marine species. This study hypothesizes that haploid ESCs from marine fish could offer unique insights and tools for genetic and virological research. To address this, we successfully established and characterized a novel haploid ESC line, hMMES1, derived from marine medaka (*Oryzias melastigma*). The hMMES1 cells contain 24 chromosomes, exhibit core stem cell characteristics, and express key pluripotency markers. In vitro, hMMES1 cells form embryonic bodies (EBs) capable of differentiating into the three germ layers. In vivo, hMMES1 cells were successfully transplanted into marine medaka and zebrafish, resulting in the generation of interspecies and interordinal chimeras. Additionally, hMMES1 cells demonstrate high efficiency in transfection and transduction, and show susceptibility to major aquaculture viruses, nodavirus (NNV) and iridovirus (SGIV). These findings suggest that hMMES1 cells represent a valuable model for genetic manipulation and virological studies in marine fish species.

## 1. Introduction

Haploid embryonic stem cells (haESCs), possessing only one set of chromosomes, have proven to be an excellent tool for genetical research and have opened up possibilities for developing novel breeding strategies through cell engineering technologies. haESCs have two distinctive features: genomic haploidy and developmental pluripotency. The genomic haploidy of haESCs offers advantages in the exploration of recessive mutations within genomes [1]. For example, haploid mammalian cells, KBM-7, were utilized for genome-wide genetic screening to identify the host factor of the bacterial toxin. This haESC screening also led to the discovery of multiple candidate receptors for the Ebola virus [2]. Similarly, mouse haESCs were employed in whole-genome genetic screening to obtain cell lines resistant to ricin, resulting in the identification of various host factors that mediating the toxicity of ricin, such as Gpr107 [3]. Another advantage of haESCs is the developmental pluripotency, which enables them to differentiate into a wide range of cell types both in vivo and in vitro. The purified haESCs were injected into blastocysts to produce chimeras, resulting in the successful transmission of marker genes through the germline to produce healthy offspring mice [4]. In the case of fish, the successful breeding of the first semi-clonal fish, named Holly, was achieved by transplanting the haploid nucleus of medaka haESCs into mature oocytes. Holly was capable of breeding normally, demonstrating the successful transfer of genetic information through haESCs and the production of fertile offspring [5]. 

The cultivation of haESCs was first achieved in Japanese medaka (*Oryzias latipes*) [5], followed by mice [3,6], monkeys [7,8], and humans [9]. Nonetheless, at present, the development of diploid fish ESCs has been confined in some fish species, such as zebrafish and medaka [10,11,12], but the derivation of fish haESCs was solely to the Japanese medaka (*O. latipes*). Until now, establishing haESCs lines within marine fish species has still been a substantial and intricate challenge. Marine medaka (*Oryzias melastigma*) is currently a widely used marine model fish. The small body size, short generation time, and year-round egg spawning of marine medaka make it an excellent candidate for marine fish genetics research and breeding studies [13,14]. Given the remarkable genetical and physiological differences between freshwater and seawater fish, coupled with the absence of haESCs in marine fish, marine medaka was selected as the preferred candidate for the development of marine fish haESCs in this study. We present the derivation and characterization of haploid ESCs (hMMES1) from marine medaka, as well as investigating their differentiation capacity and susceptibility to fish viruses. The establishment of the hMMES1 cell line will offer a valuable cellular resource for the field of marine fish genetic studies.

## 2. Materials and Methods

### 2.1. Fish and Viruses

The marine medakas (*O. melastigma*) used for this study were hatched and reared in house and maintained in the aquarium facility of the Department of Marine Sciences, Sun Yat-Sen University (Zhuhai, Guangdong, China) for more than three generations. The fish were reared under the controlled conditions of 26 °C and in artificial seawater, prepared by mixing sea salt (Red Sea Aquatics, Guangzhou, China) with a salinity of 30‰, and fed with live Artemia sp. three times a day. All animal handling and experimental procedures were approved by the Ethics Committee of Sun Yat-Sen University (Approval No. SYSU-IACUC-2021-B0978). 

Red-spotted grouper NNV (RGNNV) was isolated from diseased sea perch in Guangdong Province of China [15]. Singapore grouper iridovirus (SGIV) was obtained from the National University of Singapore [16]. The virus stock was frozen at −80 °C until use.

### 2.2. Induced Gynogenesis

A total of 30 female and 30 male fish were used for in vitro normal fertilization and gynogenetic fertilization, respectively. Haploid gynogenetic embryos of marine medaka were generated following previous reports [17]. Mature male and female fish were separated one day before the experiment. The ovaries were carefully dissected to release the mature oocytes into medaka oocyte medium (MOM) (M199 9.8 g; penicillin–streptomycin solution 100 µg–100 U mL^−1^) until use [17]. The testis was rinsed with cold phosphate-buffered saline (PBS, pH 7.2) and then dissociated into small aggregates by sharp forceps in 50 μL PBS on ice to release the sperm. The sperm was then exposed to UV light at a dose of 1250–2500 mJ (GS Gene Linker UV Chamber, Bio-Rad, Hercules, CA, USA) and immediately mixed with 10–40 eggs in MOM for gynogenetic activation in darkness. Successful induction of haploid gynogenesis was examined by haploid metaphases of embryos.

### 2.3. Cell Culture

The marine medaka embryos of natural fertilization or haploid gynogenesis were treated with proteinase K (Sigma, cat. no. P6556, Sigma-Aldrich, St. Louis, MO, USA) (10 mg/mL) at 28 °C for 90 min to remove the filaments, then rinsed twice with PBS for 2 min. After they had developed to the blastula stage, 8–12 embryos were separated for blastomeres under aseptic conditions and seeded into a gelatin-coated 48-well plate at 28 °C in ESM2 medium (DMEM 13.37 g per liter; HEPES 20 mM; L-Glutamine 2 mM; nonessential amino acid 1 mM; sodium pyruvate 1 mM; sodium selenite 2 mM; 2-Mercaptoethanol 100 µM; penicillin–streptomycin solution 100 µg–100 U mL^−1^; fetal bovine serum 15%; human recombinant bFGF 10 ng mL^−1^; medaka embryo extract 1 embryo per mL) [17]. The primary cultures were serially subcultured at a split ratio of 1:2 until the cells were capable of stable growth.

### 2.4. Cell Colony Formation

The primary cell cultures were reseeded into a gelatin-coated dish at 10^4^ cells per 10 cm dish for cell colonies growth [17]. Individual colonies were mechanically picked into gelatin-coated 96-well plates under a stereomicroscope. The fully grown colonies in 96-well plates were then transferred into 48-, 24-, 12-, and 6-well plates for expansion growth.

### 2.5. Cytogenetic Analyses

The metaphases of embryos and cultured cells were prepared and analyzed as described previously [18]. Briefly, the dechonorionated embryos or cells were firstly incubated with colchicine (Sigma, cat. no. C9754, Sigma-Aldrich, St. Louis, MO, USA) (1 μg/mL) for 2 h. After hypotonic treatment with 0.075 M KCl (Sigma, cat. no. P5405, Sigma-Aldrich, St. Louis, MO, USA), the embryo or cells was transferred into fresh fixative for 10 min, then dropped onto a cold wet slide for air-drying and chromosome staining. 

Cell cultures in 6-well plates were harvested by trypsinization for flow cytometry analysis. The testicular and liver cells of marine medaka were dissociated with PBS from mature testes and liver through dissection, respectively. These cells were then prepared for DNA staining as described previously [19]. The cell cycle was analyzed through a Coulter Elite ESP flow cytometer equipped with WinMDIv2.8 software (Beckman Coulter, West Sacramento, CA, USA). 

### 2.6. Alkaline Phosphatase (AP) Staining

Cells at 50–60% confluence in 6-well plates were washed with PBS and fixed with 500 μL of freshly mixed methanol–acetone (1:1) for 10 min. The cells were stained with BCIP/NPT solution (Beyotime) in darkness at 28 °C for 2 h and covered with 200 μL of glycerol for visualization under a microscope. 

### 2.7. Growth Curves

Cell growth at different fetal bovine serum (FBS, Invitrogen, Carlsbad, CA, USA) concentrations and temperatures was analyzed. Briefly, 3 × 10^4^ cells were incubated at 28 °C in ESM4 with 10%, 15%, and 20% FBS for cell number counting in 5 days, respectively. At 20 °C, 28 °C, and 32 °C, the cells were incubated in ESM4 with 15% FBS for cell number counting in 5 days, respectively. Each experiment was carried out in triplicate.

### 2.8. RNA Extraction and qPCR Analysis 

Total RNA of the cells was extracted using the RNA extraction kit and RNAiso reagent (TaKaRa Bio Inc., Kusatsu, Japan). cDNA was synthesized using PrimeScript Reverse Transcriptase (TaKaRa Bio Inc., Kusatsu, Japan) with 1 μg of total RNA according to the manufacturer’s instructions. qPCR was performed in a LightCycler 480 Ⅱ (Roche, Basel, Switzerland) system with the follow cycling conditions: 95 °C for 30 s, 45 cycles of 95 °C for 15 s, 60 °C for 15 s, and 72 °C for 15 s, followed by melting curve analysis to verify the specificity of the amplified products. The sequences of primers used are listed in Supplementary Table S1. The mean Cq values of each sample were derived from triplicated experiments and calculated through the 2^−ΔΔCt^ method. Marine medaka *β-actin* was used as the reference. The expression of targeted genes, including *oct4*, *klf4*, *myc*, *sall4*, *tcf3a*, *nf200*, *actinin2*, *ntl*, *sox1*, *sox10*, and *myoD*, were analyzed.

### 2.9. Embryoid Body (EB) Formation 

Cells in 6-well plate were trypsinzed for suspension culture and treated with 10 μM all-trans retinoic acid (RA; Sigma, cat. no. R2625, Sigma-Aldrich, St. Louis, MO, USA). Every 2 days, half of the medium was refreshed, with the final concentration of RA unchanged. The EBs in 10 days were collected for qPCR or dissociated into single cells in gelatin-coated plates for differentiation and further qPCR detection. 

### 2.10. Cell Transplantation

The blastula-stage embryos of marine medaka were dechorionated in proteinase K (10 mg/mL) at 28 °C for 2 h and in hatching enzyme at 28 °C for 30 min. Zebrafish embryos at the blastula stage were dechorionated using pronase E (1 mg/mL; Sigma-Aldrich, St. Louis, MO, USA) at 26 °C for 5 min. The dechorionated embryos were then rinsed in balanced-salt saline–PEG solution within 2 h for use [17].

The cultured marine medaka cells were transfected with pEGFP-N3 or pDsRED-N1 using Lipofectamine 8000 (Beyotime, Shanghai, China) following the manufacturer’s instructions. Stable GFP or DsRED protein-expressing cells were purified by G418 (1 mg/mL) treatment, followed by clonal growth and cell expansion. The GFP or DsRed labelled cells were suspended in cell transplantation medium (100 mM NaCl, 5 mM KCl, 5 mM Hepes, pH 7.2), and injected into dechorionated marine medaka or zebrafish blastula embryos at about 100 cells per embryo.

### 2.11. Cell Transfection and Transduction

pEGFP-N3 plasmid was transfected into MMES1 and hMMES1 cells in six-well plates (1 × 10^6^ cells/well) by using Lipofectamine 8000. MMES1 and hMMES1 cells were infected with retrovirus (rvLcherry) [20] by mixing 10^6^ cells, retrovirus, polybrene (8 μg/mL, Sigma-Aldrich, St. Louis, MO, USA), and ESM4 medium at room temperature for 2 h. The cells were then subjected to centrifugation at 2000 rpm, followed by seeding onto 6-well plates with 2 mL of ESM4 per well. The percentage of cells that were positive for GFP or cherry was monitored by fluorescent microscopy at 2 days following transfection or infection and determined by cell counting to evaluate the transfection or transduction efficiency.

### 2.12. Statistics

All statistical data in this study were analyzed using SPSS version 20.0 and presented as the mean ± standard error of the mean for experiments conducted with at least three independent experiments. Data were statistically analyzed using either Student’s *t*-test for two-group comparisons or one-way ANOVA for multiple-group comparisons. *p* < 0.05: statistically significant difference; *p* < 0.01: highly significant difference.

## 3. Results 

### 3.1. Derivation of Marine Medaka Haploid ESCs

**Figure 1:**
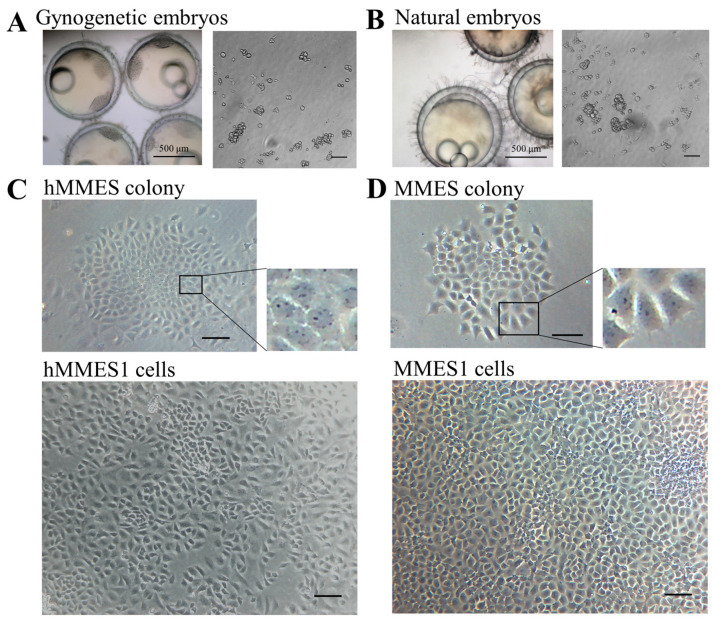
The derivation of marine medaka ESCs. (**A**) Marine medaka gynogenetic embryos and gynogenetic blastomeres. (**B**) Marine medaka normal embryos and their blastomeres. (**C**) hMMES cell colony and hMMES1 cells at passage 30. (**D**) MMES cell colony and MMES1 cells at passage 30. Scale bars, 200 μm unless otherwise indicated. All experiments were performed at least three times, and representative data are shown.

Gynogenetic embryos of marine medaka (Figure 1A) were used for haploid ESC derivation, with naturally fertilized embryos (Figure 1B) as a control. As shown in Figure 1A,B, cells from embryo blastomeres attached to the plates after 24 h culture and grew to be fully confluent within 3–5 days. The cells were subcultured at a split ratio of 1:1 for the first time, followed by ratios of 1:2 or 1:3 ratio for subsequent passages. Morphologically, the cells derived from gynogenetic embryos displayed a round or polygonal morphological phenotype, characterized by a small cytoplasm, large nuclei, and prominent nucleoli. Following colony formation and subsequent subculturing over 50 times within 4 months, these colonies were identified as haploid MMES, with a success rate of 58.3% (with hMMES1 representing a specific example) (Figure 1C). The cells originating from naturally fertilized embryos were labeled as MMES1 and served as the control (Figure 1D).

### 3.2. Karyotype, Pluripotency, and Growth Kinetics of hMMES1 Cells 

**Figure 2:**
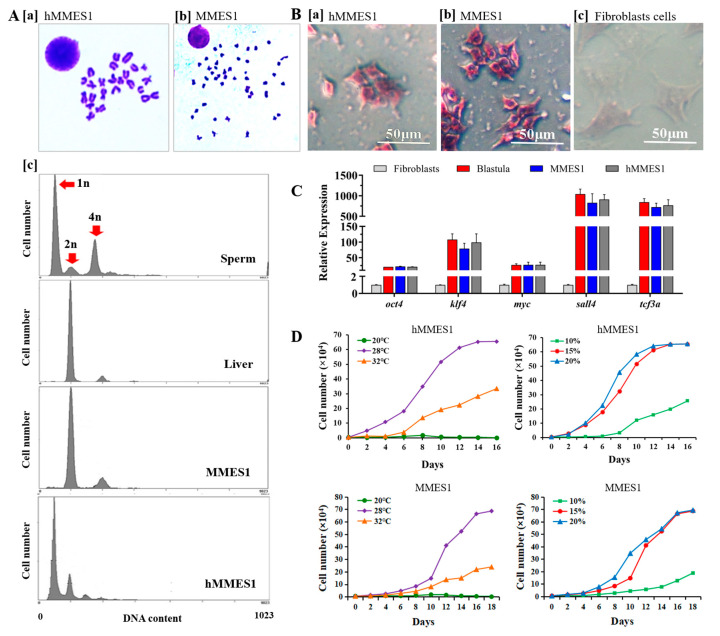
Karyotype, pluripotency, and growth curves of hMMES1. (**A**) Chromosome metaphase of hMMES1 [**a**] and MMES1 [**b**], respectively; [**c**] flow analyses of DNA content in hMMES1, MMES1, sperm, and liver cells from marine medaka. (**B**) Alkaline phosphatase staining of hMMES1 [**a**] and MMES1 [**b**] at day 248 of culture; the cultured fibroblasts cells [**c**] were stained as a control. (**C**) Expression of pluripotency marker genes in hMMES1, MMES1, and fibroblast cells by qRT-PCR; *β-actin* was used as a reference gene. Data are representative of three repeated experiments as mean ± SEM. One-way ANOVA was used for multiple-group comparisons. (**D**) Growth curves of hMMES1 and MMES1 cells at different temperatures in ESM4 supplemented with 15% fetal bovine serum or different concentrations of FBS at 28 °C. Representative data from three independent experiments are presented.

The karyotype analysis revealed a modal chromosome number of 24 for hMMES1 (Figure 2(A[a])) and 48 chromosomes for MMES1 cells (Figure 2(A[b])). Flow cytometry analysis showed hMMES1 cells had a haploid DNA content similar to sperm cells, while MMES1 cells had a DNA content similar to diploid liver cells (Figure 2(A[c])). 

The pluripotency of ESCs is typically characterized by high activity of alkaline phosphatase (AP) and the expression of pluripotent marker genes [5]. As shown in Figure 2(B[a–c]), both hMMES1 and MMES1 cells exhibited high AP activity, as indicated by the dark staining of AP. qRT-PCR analysis showed that the key pluripotency genes, including *oct4*, *klf4*, *myc*, *sall4*, and *tcf3a*, were highly expressed in hMMES1 and MMES1 cells (Figure 2C), implying these cells possessed developmental pluripotency.

Cell growth kinetics analysis showed hMMES1 cells exhibited the maximum growth rate in ESM4 containing 20% FBS at 28 °C, which is similar to MMES1 cells (Figure 2D). After cryopreservation at −196 °C for 1 to 3 months, hMMES1 and MMES1 cells could be successfully recovered with approximately 75–80% viability.

### 3.3. Induced Differentiation of hMMES1 Cells In Vitro and In Vivo

**Figure 3:**
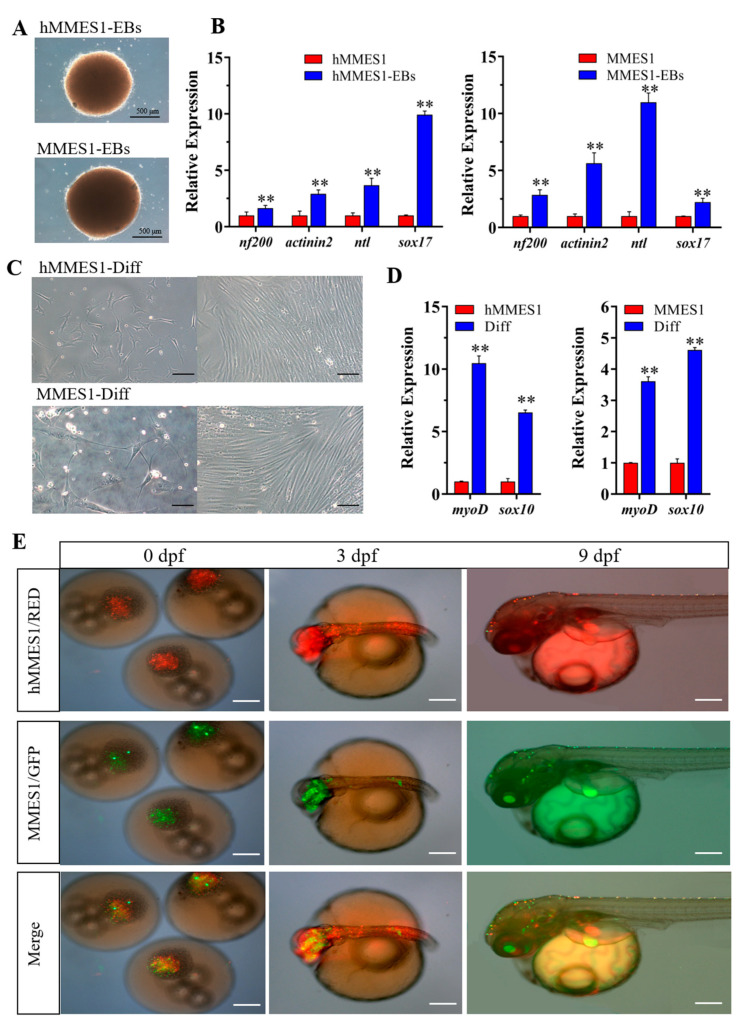
The formation and differentiation of hMMES1-derived embryoid bodies (EBs). (**A**) Morphology of 10-day-old EBs derived from hMMES1 and MMES1. (**B**) Expression analysis of gene markers specific to the ectoderm (*nf200*), mesoderm (*actinin2* and *ntl*), and endoderm (*sox17*) in 10-day-old hMMES1 or MMES1-derived EBs by qRT-PCR. Data are presented as the means ± SD of three independent experiments. Student’s *t*-test was conducted for two-group comparisons (** *p* < 0.01). (**C**) Morphology of differentiated cells from hMMES1- or MMES1-derived EBs. (**D**) Expression analysis of muscle (*myoD*) and neural (*sox10*) marker genes expression in hMMES1 or MMES1 differentiated cells by qRT-PCR. Data are represented as mean ± SEM from three independent replicates. Student’s *t*-test was conducted for two-group comparisons (** *p* < 0.01). (**E**) Micrograph of marine medaka chimera embryo host (*n* = 120), showing transplanted hMMES1 (red) or MMES1 (green) at 0, 3, and 9 days post-fertilization (dpf). Scale bars, 100 μm. Representative data are shown from three independent experiments.

ESCs possessed the proficiency to generate three-dimensional embryoid bodies (EBs) in suspension culture environments and had the capabilities of induced differentiation [5]. As shown in Figure 3A, hMMES1-derived EBs were synthesized in vitro after a 10-day period of suspension culture, paralleling the MMES1-derived EBs. qPCR analysis revealed these EBs highly expressed the marker genes specific to three germ layers, including the ectoderm (*nf200*), the mesoderm (*actinin2* and *ntl*), and the endoderm (*sox17*) (Figure 3B). Furthermore, the differentiation potential of the EBs was assessed by attachment cell culture. hMMES1- and MMES1-derived EBs were observed to differentiate into several cell types (Figure 3C), accompanied by an upregulation in the expression levels of specific markers for neurons (*sox10*) and muscle (*myoD*) (Figure 3D). These results demonstrated the in vitro differentiation capability of hMMES1 cells.

The pluripotency of hMMES1 cells in vivo was tested by chimera formation in marine medaka. RFP-labeled hMMES1 cells were transplanted into marine medaka blastulas, which resulted in an 80% survival rate (Figure 3E), and GFP-labeled MMES1 cells were transplanted as a control. At 3 days post-fertilization (dpf) and 9 dpf, RFP-labeled hMMES1 cells were observed in chimera survivors, exhibiting a similar distribution of fluorescence with MMES1 cells in head neural, fin epithelium, and the heart. These results confirmed that hMMES1 cells were able to participate in the chimeric embryogenesis through transplantation.

### 3.4. Interordinal Chimera Formation of hMMES1 Cells in Zebrafish

**Figure 4:**
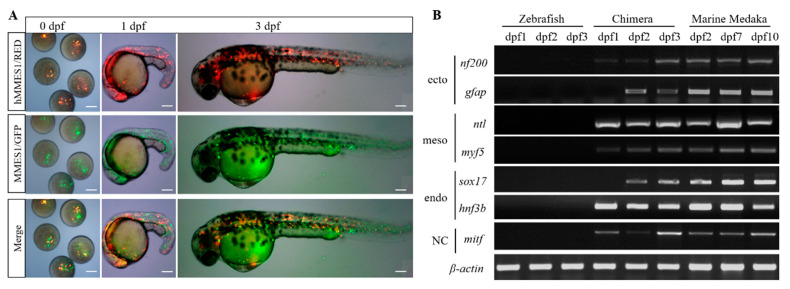
Interspecific chimera formation of hMMES1 in a zebrafish host. (**A**) Micrograph of zebrafish embryo host (*n* = 200) showing transplanted hMMES1 (red) and MMES1 (green) at 0, 1, and 3 dpf. Scale bars, 100 μm. (**B**) Molecular analyses of zebrafish transplanted with MMES1 and hMMES1, wild zebrafish, and medaka embryos at day 1–10 (D1–D10) post-fertilization. The ectoderm markers (*nf200* and *gfap*), mesoderm markers (*ntl* and *myf5*), endoderm markers (*hnf3b* and *sox17*), and neural crest markers (*mitf*) were amplified by using primers specific to medaka cDNAs; *β-actin* of marine medaka or zebrafish was amplified as a reference. All experiments were performed three times, and representative data are shown. The uncropped nucleic acid gels figures are presented in the Supplementary Materials.

Interspecific chimera formation has been widely used to assess the differentiation potential of stem cells [21]. Here, zebrafish embryos were transplanted with hMMES1 cells, resulting in a chimeric survival rate of 79.2%. At 2 dpf, the labeled donor cells of hMMES1 were observed in various locations, including the beating heart, eyes, and fins (Figure 4A). To further investigate the differentiation of donor cells in a heterologous zebrafish host, the expression of six marine medaka lineage molecular markers (*nf200*, *gfap*, *ntl*, *myf5*, *sox17*, *hnf3b*, and *mitf*) was analyzed (Figure 4B). RT-PCR analysis revealed that the expression of *gfap* and *sox17* in an interspecific marine medaka–zebrafish chimera was not detected at 1 dpf but became detectable at 2–3 dpf. The expression of other genes was observed throughout the three-day period.

### 3.5. Cell Transfection and Transduction Efficiency 

**Figure 5:**
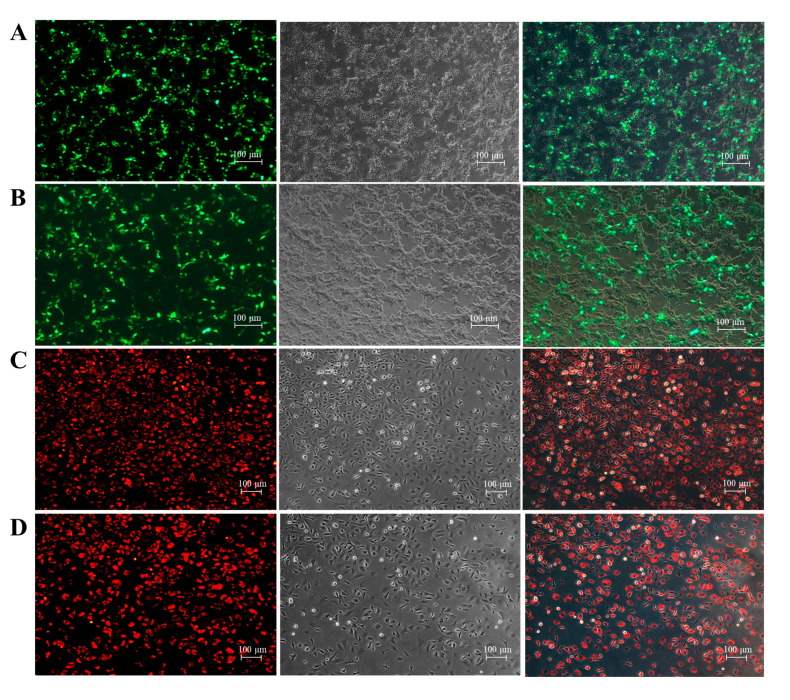
Transfection and transduction efficiency of hMMES1 cells. (**A**,**B**): hMMES1 (**A**) and MMES1 (**B**) cells were transfected with pEGFP-N3 plasmid for 48 h. Bar = 100 μm. (**C**,**D**): hMMES1 (**C**) and MMES1 (**D**) cells were infected with rvLcherry at MOI = 50 for 48 h. Bar = 100 μm. Representative images of cells are shown from three independent experiments.

The transfection efficacy of hMMES1 cells was assessed via the introduction of GFP plasmids. Green fluorescence emissions were initially observable at 24 h after transfection, culminating in an estimated transfection efficiency of approximately 40% at 48 h (Figure 5A), which was similar with MMES1 cells (Figure 5B). Following retrovirus infection, both hMMES1 and MMES1 cells exhibited a transduction efficiency exceeding 80%, as evidenced by the presence of cherry fluorescence signaling (Figure 5C,D). These findings indicated that the hMMES1 cell line is an optimal candidate for external gene manipulation.

### 3.6. Viral Susceptibility of hMMES1 Cells

**Figure 6:**
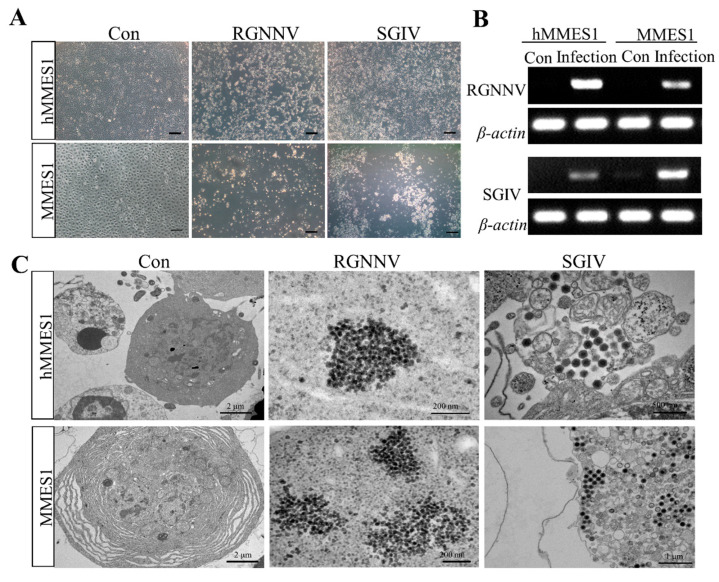
Viral susceptibility of hMMES1 cells. (**A**) hMMES1 and MMES1 cells were infected with RGNNV or SGIV for 48 h. Bar = 100 μm. (**B**) Expression analysis of RGNNV or SGIV mRNA in hMMES1 and MMES1 cells at 48 h following virus infection. *β-actin* of marine medaka was amplified as a reference. All experiments were performed three times and shown with the representative data. (**C**) Transmission electron micrograph (×50,000) of RGNNV- or SGIV-infected hMMES1 or MMES1 cells; cells treated with PBS were used as a control (Three independent biological experiments, *n* = 3). The uncropped nucleic acid gels figures are presented in the Supplementary Materials.

RGNNV and SGIV are two major viral pathogens that pose threats to marine fish, resulting in substantial economic losses [22,23]. To investigate the susceptibility of marine medaka ESCs to these viruses, hMMES1 and MMES1 cells were infected with RGNNV and SGIV. As shown in Figure 6A, RGNNV- or SGIV-infected hMMES1 cells exhibited significant cytopathic effects at 24 hpi, including cell rounding and detachment. RT-PCR analysis confirmed the presence of partial fragments of the RGNNV and SGIV genes in the infected hMMES1 cells as well as MMES1 cells (Figure 6B). Furthermore, transmission electron microscopy revealed the presence of several RGNNV and SGIV particles in the cytoplasm of hMMES1 or MMES1 cells following RGNNV or SGIV infection (Figure 6C). 

## 4. Discussion 

The utilization of haploid ESCs in genetic screening has established a robust foundation for mouse and human developmental and reproductive biology [24]. However, in comparison to mammals, the development and application of haploidy in fish species significantly lags behind. Although the successful derivation of haploid pluripotent ESCs from Japanese medaka has been previously achieved [5], the generation of haploid ESCs in other fish species remains lacking to date. The marine medaka (*O. melastigma*), with diminutive size, brief generational span and rich genetic resources, stands out as an advantageous model for genetic and breeding research in marine fish, distinguishing itself from freshwater models like the zebrafish (*Danio rerio*) and the freshwater medaka (*O. latipes*) (Kim et al., 2018). In this study, the haploid ESC line (hMMES1) from marine medaka, the marine model fish, was derived; it not only retained pluripotency but also exhibited high transfection and transduction efficiency, along with a susceptibility to RGNNV and SGIV. The hMMES1 cell line thus marks a pioneering advancement as the next fish haploid ESCs after the Japanese medaka and, distinctively, as the first haploid ESCs within a marine fish species.

Haploid ESCs, combining haploidy and pluripotency, have rapidly emerged as a practical and effective tool for studying gene functions. Significantly, the haploid constitution confers marked benefits in genetic screening over diploidy by obviating the concealment of mutational impacts, attributable to the genomic copy [25]. Recently, haploid cells have been extensively utilized to decipher pivotal cellular factors and pathways critical for pathogen interaction. For example, haploid cells have been instrumental in unearthing host factors pivotal for the influenza virus [2], Ebola virus [26], and cholera toxin [27]. Here, hMMES1 cells were developed, embodying haploidy and pluripotency, evidenced by their 24 chromosomes, and their capacity to endure, proliferate, and their embryogenesis post-transplantation. This aligns with previous findings where haploid ESCs were shown to be effective in genetic screenings. Given the haploid nature of hMMES1 cells, they were subjected to screenings for viral receptors, which successfully pinpointed HSP90ab1 as a critical receptor for NNV [28]. These findings highlight the potential of hMMES1 cells as a valuable tool in genetic screening and in pioneering gene-editing techniques.

The capacity to differentiate has been widely regarded as the fundamental basis for the genetic and breeding utilization of ESCs. On that basis, the interspecific chimera formation has emerged as a more critical option for testing the pluripotency of stem cells in vivo [29,30]. Research involving medaka and zebrafish illustrated that medaka ESCs are capable of aligning with the developmental trajectory of zebrafish, thereby underscoring the viability of interspecific chimera formation [21]. Another breakthrough in transplantation across different fish species was achieved through the successful transplantation of sea perch–zebrafish chimeras [31]. In the current study, we embarked on transplanting hMMES1 cells into zebrafish blastulas, culminating in the successful production of marine medaka–zebrafish chimeras. This achievement further supports the pluripotency of hMMES1 cells and demonstrates their potential utility in generating genetically manipulated organisms across species. Our findings extend the previous work on cross-species chimera formation and highlight the versatility of marine medaka ESCs in transplantation and developmental studies.

Viral infections have caused severe economic losses in both marine and freshwater fish. To understand the pathogenesis of these viruses, it is crucial to establish susceptible and resistant cell lines. Here, we examined the susceptibility of marine medaka ESC cells to two highly infectious and notable fish viruses, RGNNV and SGIV. RGNNV, classified as a betanodavirus, is notorious for its precipitous mortality rates (80–100%) in fish, predominantly affecting hatchery-bred larvae and juveniles [32]. SGIV, categorized under the genus Ranavirus, is a virulent DNA virus responsible for severe systemic afflictions and extensive losses in marine aquaculture [23]. Previous research has identified fish cell lines susceptible to these viruses, such as the TOK cell line from golden pompano [33] and the GS cells from orange-spotted grouper [34]. Notably, these viral pathogens not only infect fish larvae but also target fish embryos, which facilitate viral vertical transmission. Research has demonstrated the capability of RGNNV for replication across various developmental stages of fish embryos [35], elucidating the frequent detection of betanodavirus in both fish embryos and larvae [36]. Hence, it is of critical importance to explore RGNNV and SGIV infections and defense mechanisms in embryonic cells. Until now, multiple cell lines derived from fish embryos, such as those obtained from grouper [16], zebrafish [37], and medaka [38], have been established and demonstrated susceptibility to RGNNV or SGIV. Yet, the sensitivity of ESCs derived from marine fish to these viruses has remained uncharted. Our study builds on this body of work by exploring the susceptibility of ESCs derived from marine fish. We observed typical CPEs, viral gene expression, and viral particles in both RGNNV- and SGIV-infected hMMES1 and MMES1 cells, demonstrating the high susceptibility of marine medaka ESCs to these viruses. These findings parallel previous studies on other fish cell lines, confirming that hMMES1 cells provide a valuable platform for viral isolation, propagation, and understanding the pathogenesis of fish viral pathogens. This discovery opens new avenues for studying viral infections in marine species, offering the potential for significant advances in aquaculture disease management.

## 5. Conclusions

In summary, the haploid ESCs, designated as hMMES1, have been successfully derived and characterized from marine medaka. The hMMES1 cells possess various cellular properties, including an ES cell phenotype, pluripotency gene expression, and the capacity for differentiation both in vitro and in vivo. Furthermore, hMMES1 cells exhibit high efficiency of transfection and transduction, coupled with a notable susceptibility to viral infections. Consequently, the hMMES1 cell line represents an invaluable asset for advancing research in marine fish genetics and virology.

## Data Availability

All data included in this study are available upon request from the corresponding author.

## Supplementary Materials

The following supporting information can be downloaded at: https://www.mdpi.com/article/10.3390/ani14182739/s1. Table S1. The sequence and PCR efficiency of primers used in this study. The uncropped nucleic acid gels figures.

## References

[1] Jin L.F., Li J.S. (2016). Generation of genetically modified mice using CRISPR/Cas9 and haploid embryonic stem cell systems. Zool. Res..

[2] Carette J.E., Guimaraes C.P., Varadarajan M., Park A.S., Wuethrich I., Godarova A., Kotecki M., Cochran B.H., Spooner E., Ploegh H.L. (2009). Haploid genetic screens in human cells identify host factors used by pathogens. Science.

[3] Elling U., Taubenschmid J., Wirnsberger G., O’Malley R., Demers S.-P., Vanhaelen Q., Shukalyuk A.I., Schmauss G., Schramek D., Schnuetgen F. (2011). Forward and reverse genetics through derivation of haploid mouse embryonic stem cells. Cell Stem Cell.

[4] Leeb M., Walker R., Mansfield B., Nichols J., Smith A., Wutz A. (2012). Germline potential of parthenogenetic haploid mouse embryonic stem cells. Development.

[5] Yi M., Hong N., Hong Y. (2009). Generation of medaka fish haploid embryonic stem cells. Science.

[6] Leeb M., Wutz A. (2011). Derivation of haploid embryonic stem cells from mouse embryos. Nature.

[7] Li W., Li X., Li T., Jiang M.G., Wan H., Luo G.Z., Feng C., Cui X., Teng F., Yuan Y. (2014). Genetic modification and screening in rat using haploid embryonic stem cells. Cell Stem Cell.

[8] Yang H., Liu Z., Ma Y., Zhong C., Yin Q., Zhou C., Shi L., Cai Y., Zhao H., Wang H. (2013). Generation of haploid embryonic stem cells from *Macaca fascicularis* monkey parthenotes. Cell Res..

[9] Sagi I., Chia G., Golan-Lev T., Peretz M., Weissbein U., Sui L., Sauer M.V., Yanuka O., Egli D., Benvenisty N. (2016). Derivation and differentiation of haploid human embryonic stem cells. Nature.

[10] Lee D., Ryu J.H., Lee S.T., Nam Y.K., Kim D.S., Gong S.P. (2015). Identification of embryonic stem cell activities in an embryonic cell line derived from marine medaka (*Oryzias dancena*). Fish Physiol. Biochem..

[11] Zilova L., Weinhardt V., Tavhelidse T., Schlagheck C., Thumberger T., Wittbrodt J. (2021). Fish primary embryonic pluripotent cells assemble into retinal tissue mirroring in vivo early eye development. Elife.

[12] Son M.J., Gong S.P. (2022). Feeder cell-dependent primary culture of single blastula-derived embryonic cell lines from marine medaka (*Oryzias dancena*). In Vitro Cell. Dev. Biol. Anim..

[13] Kim B.M., Kim J., Choi I.Y., Raisuddin S., Au D.W., Leung K.M., Wu R.S., Rhee J.S., Lee J.S. (2016). Omics of the marine medaka (*Oryzias melastigma*) and its relevance to marine environmental research. Mar. Environ. Res..

[14] Ye R.R., Lei E.N., Lam M.H., Chan A.K., Bo J., van de Merwe J.P., Fong A.C., Yang M.M., Lee J.S., Segner H.E. (2011). Gender-specific modulation of immune system complement gene expression in marine medaka Oryzias melastigma following dietary exposure of BDE-47. Environ. Sci. Pollut. Res. Int..

[15] Jia P., Jia K.T., Yi M.S. (2015). Complete genome sequence of a fish nervous necrosis virus isolated from Sea perch (*Lateolabrax japonicus*) in China. Genome. Announc..

[16] Liu Y., Tran B.N., Wang F., Ounjai P., Wu J., Hew C.L. (2016). Visualization of assembly intermediates and budding vacuoles of singapore grouper iridovirus in grouper embryonic cells. Sci. Rep..

[17] Yi M., Hong N., Hong Y. (2010). Derivation and characterization of haploid embryonic stem cell cultures in medaka fish. Nat. Protoc..

[18] Le Y., Li Y., Jin Y., Jia P., Jia K., Yi M. (2017). Establishment and characterization of a brain cell line from sea perch, *Lateolabrax japonicus*. In Vitro Cell. Dev. Biol. Anim..

[19] Zhang W., Jia P., Liu W., Jia K., Yi M. (2019). Screening for antiviral medaka haploid embryonic stem cells by genome wide mutagenesis. Mar. Biotechnol..

[20] Liu Q., Wang Y., Lin F., Zhang L., Li Y., Ge R., Hong Y. (2015). Gene transfer and genome-wide insertional mutagenesis by retroviral transduction in fish stem cells. PLoS ONE.

[21] Hong N., Chen S., Ge R., Song J., Yi M., Hong Y. (2012). Interordinal chimera formation between medaka and zebrafish for analyzing stem cell differentiation. Stem Cells Dev..

[22] Bandín I., Souto S. (2020). Betanodavirus and VER Disease: A 30-year Research Review. Pathogens.

[23] Yuan Y., Hong Y. (2016). Subcellular redistribution and sequential recruitment of macromolecular components during SGIV assembly. Protein Cell.

[24] Yilmaz A., Peretz M., Sagi I., Benvenisty N. (2016). Haploid human embryonic stem cells: Half the genome, double the value. Cell Stem Cell.

[25] Pillay S., Carette J.E. (2015). Hunting viral receptors using haploid cells. Annu Rev Virol.

[26] Carette J.E., Raaben M., Wong A.C., Herbert A.S., Obernosterer G., Mulherkar N., Kuehne A.I., Kranzusch P.J., Griffin A.M., Ruthel G. (2011). Ebola virus entry requires the cholesterol transporter Niemann-Pick C1. Nature.

[27] Guimaraes C.P., Carette J.E., Varadarajan M., Antos J., Popp M.W., Spooner E., Brummelkamp T.R., Ploegh H.L. (2011). Identification of host cell factors required for intoxication through use of modified cholera toxin. J. Cell Biol..

[28] Zhang W., Jia K., Jia P., Xiang Y., Lu X., Liu W., Yi M. (2020). Marine medaka heat shock protein 90ab1 is a receptor for red-spotted grouper nervous necrosis virus and promotes virus internalization through clathrin-mediated endocytosis. PLoS Pathog..

[29] Chen J.J., Lei K. (2023). The known, unknown, and unknown unknowns of cell-cell communication in planarian regeneration. Zool. Res..

[30] Guo L.Y., Wei J.K., Yang S.C., Wang Z.B. (2012). Glaucoma model for stem cell transplantation research in New Zealand white rabbits. Zool. Res..

[31] Chen S.-L., Sha Z.-X., Ye H.-Q. (2003). Establishment of a pluripotent embryonic cell line from sea perch (*Lateolabrax japonicus*) embryos. Aquaculture.

[32] Zhang W.W., Jia P., Lu X.B., Chen X.Q., Weng J.H., Jia K.T., Yi M.S. (2022). Capsid protein from red-spotted grouper nervous necrosis virus induces incomplete autophagy by inactivating the HSP90ab1-AKT-MTOR pathway. Zool. Res..

[33] Zhou L., Li P., Liu J., Ni S., Yu Y., Yang M., Wei S., Qin Q. (2017). Establishment and characterization of a mid-kidney cell line derived from golden pompano *Trachinotus ovatus*, a new cell model for virus pathogenesis and toxicology studies. In Vitro Cell. Dev. Biol. Anim..

[34] Qin Q.W., Wu T.H., Jia T.L., Hegde A., Zhang R.Q. (2006). Development and characterization of a new tropical marine fish cell line from grouper, Epinephelus coioides susceptible to iridovirus and nodavirus. J. Virol. Methods.

[35] Kuo H.C., Wang T.Y., Hsu H.H., Chen P.P., Lee S.H., Chen Y.M., Tsai T.J., Wang C.K., Ku H.T., Lee G.-B. (2012). Nervous necrosis virus replicates following the embryo development and dual infection with iridovirus at juvenile stage in grouper. PLoS ONE.

[36] Gomez D.K., Sato J., Mushiake K., Isshiki T., Okinaka Y., Nakai T. (2004). PCR-based detection of betanodaviruses from cultured and wild marine fish with no clinical signs. J. Fish Dis..

[37] Wang F., Zhu Y., Hew C.L. (2015). Quantitative study of proteomic alterations in a zebrafish (*Danio rerio*) cell line infected with the singapore grouper iridovirus (SGIV). Virus Res..

[38] Yuan Y., Huang X., Zhang L., Zhu Y., Huang Y., Qin Q., Hong Y. (2013). Medaka haploid embryonic stem cells are susceptible to Singapore grouper iridovirus as well as to other viruses of aquaculture fish species. J. Gen. Virol..

