# Supplementary material for: Characterizing Marine Medaka (Oryzias melastigma) Haploid Embryonic Stem Cells: A Valuable Tool for Marine Fish Genetic Research
Source: Animals (Basel). 2024 Sep 21;14(18):2739. doi: 10.3390/ani14182739 (PMC11428384; doi:10.3390/ani14182739)
Supplement: Supplementary file 1 [file animals-14-02739-s001.zip › animals-3190953-supplementary.pdf]

S1 Table. The sequence and PCR efficiency of primers used in this study.

Genes	primer 5’	primer 3’
**β-actin**	ATGCCCCTCGTGCTGTTT	TCTCTGTTGGCTTTGGGATTC
**oct4**	CATCCACGCAGGGCTTTT	TCTCGGGTCTGGGCTATTG
**klf4**	AAGAGGGTGGCGACGCA	AACGTGCAAACTTCCAGCC
**myc**	GGAGACGCCCAAGCAGA	ACTTGCGGTTGCTGCTGAT
**sall4**	CTA AAGTGGCTGTYTCCCA	TGAATCTGGAGGGCACTGT
**tcf3a**	ATCAACGAGGCCTTCAAGGAG	CATGTGACCAACTGGGTTGTG
**nf200**	AACCTGCCGCAGCCAAAGAAC	GATTTAGGCACAGGCTTCTCTG
**actinin2**	GTATAATGATTCTGGTGGATCC	CATAGAGGGCAGTAGAGAATGC
**ntl**	ATGAGCGCGTCGAACCCGGAC	AGACGGGCGCTTTCATCCAGT
**sox17**	ATGAGTAGTCCCGATGCGGG	GCCGGAGTCCAGCCTCTTAAT
**hnf3b**	ATGATGCTTGGAGCAGTTAA	CTACGAGGAGTTCATGATTG
**mitf**	CCATGTTGGAGATGTTGGAAT	GGACAGCGTTAGTCCTTGATT
**myoD**	TGTGACCTTGACGCTCTCC	TGCTCTCCTCTCCTTCCAGTAA
**sox10**	AACGTGGACATTGGAGAGATG	GCCTGCAGAGGCCGCCTCTGC
**gfap**	TGTGACCTTGACGCTCTCC	TGCTCTCCTCTCCTTCCAGTAA
**myf5**	CTGCGGGAGCAGGTGGAGAA	GGAGGACAGGCGGTCAACAA
**RGNNV**	TACGCAAAGGTGAGAAGAA	CACAGGAGTATCAGCCGA
**SGIV**	CACGTGTCAGCGTTATGGCGAT	GTGATATCCTCATACTCTGGAC
